# Novel Technique Shows When the Fertilized Egg’s Genome Comes into Its Own

**DOI:** 10.1371/journal.pbio.0050131

**Published:** 2007-04-24

**Authors:** Liza Gross

You might say that a mother’s influence begins even before her offspring were just a twinkle in dad’s eye. During egg cell development (called oogenesis), the mother deposits gene products—consisting largely of messenger RNAs (mRNAs) and some proteins—into the developing egg. These mRNAs take over after fertilization to orchestrate the earliest stages of embryogenesis. As the embryo develops, the fertilized egg (or zygote) cuts the apron strings as it activates its own genome—which contains both maternal and paternal genes—and relies less on the pre-existing maternal mRNAs.

To navigate this maternal-to-zygotic transition (MZT) successfully, the zygote must integrate signals that control pre-existing maternal mRNA transcripts with those that activate the zygotic genome. How the embryo manages this phased transition from one class of regulatory mechanisms to another has long remained obscure.

In a new study, Stefano De Renzis, Eric Wieschaus, and colleagues describe an innovative experimental approach to determine the maternal-versus-zygotic provenance of mRNA transcripts during MZT in the fruit fly*Drosophila melanogaster*. By matching gene transcripts with their corresponding DNA template across the genome, the researchers identified classes of zygotically and maternally expressed genes and show that the embryo combines (zygotic) transcription with (maternal) message degradation to produce the localized patterns of gene expression required for organizing the early embryo.

The early zygote consists of a single membrane filled with nuclei that go through repeated rounds of division before a membrane encases each nucleus to form discrete cells. Maternal gene products direct embryonic development until nuclear division 13, when the zygotic genome assumes control.

The details of zygotic genome activation have remained elusive partly because maternal transcript degradation proceeds alongside zygotic gene transcription. Investigators have traditionally classified increased (or up-regulated) transcripts as zygotic (assuming that a jump in mRNA signals increased transcription), stable transcripts as maternal, and down-regulated transcripts as degraded maternal transcripts. Because this interpretation doesn’t account for concomitant degradation and transcription—mRNA levels can drop or stay the same even with a zygotically active genome if maternal transcripts disappear at the same time—De Renzis et al. developed an approach to distinguish the two mechanisms clearly.

They first measured gene expression levels at 1-hour intervals over the first 3 hours of embryogenesis, starting with the unfertilized egg (0–1 hour) through nuclear cycle 14 (2–3 hours), just after the zygotic genome takes over (called the midblastula transition). By the 14th nuclear cycle, many of the transcripts had either increased or decreased in abundance—but are these changes the result of transcription, degradation, or both?

**Figure:**
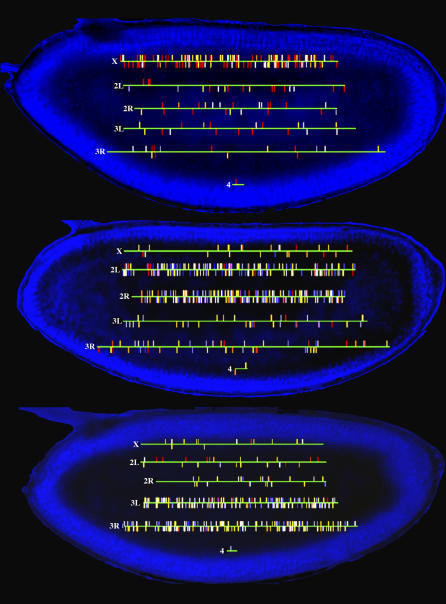
Each of these Drosophila embryos lacks one of the three major fly chromosomes: the X (top), second (middle), and third (bottom). Microarray analysis of the embryos' gene expression profile at the onset of zygotic transcription revealed which regulatory modes—transcriptional versus post-transcriptional—were at work during animal development.

Fruit fly embryos can survive the loss of entire chromosomes (long enough to study the MZT, that is) and display distinct morphologies when parts of their chromosomes are removed, allowing the researchers to see the onset of zygotic activation. They started by using fly stocks with deletions in the left arm of the second chromosome (2L)—which accounts for roughly 20% of the genome—then compared mRNAs from these embryos with those with intact 2L. Of the transcripts with reduced levels in the mutant embryos, roughly 90% correspond to genes located on 2L: deleting the arm removed the DNA templates required to make the transcripts, ergo the mRNAs are derived from zygotic transcription. Those transcripts corresponding to 2L genes that were detected in cycle 14 embryos, the researchers concluded, did not depend on 2L and thus must be the products of maternal transcription.

Next, the researchers analyzed mRNA profiles in embryos missing either the entire second chromosome, the third chromosome, or the X chromosome. Since flies have only three major chromosomes, they could distinguish between zygotic and maternal contributions across the entire genome. They identified mRNAs whose levels were directly related to the presence of each chromosome and classified these transcripts as zygotically derived.

To chart the fate of maternal transcripts, the researchers compared mRNA levels from early untreated embryos (whose transcripts are essentially maternal) with those from embryos missing one of the three chromosomes. The presence of about 30% of maternal transcripts was significantly reduced by cycle 14. For about a third of these cases, zygotic transcription compensated for the degraded transcripts, keeping expression levels at cycle 14 fairly constant, and demonstrating the pitfalls of using transcription level changes alone as an indication of zygotic activity.

About a third of the genes identified as zygotic have no maternal counterparts (most of these genes are transcription factors), but the rest, which coexist with maternal transcripts, have no particular functional bias. The newly transcribed genes may fulfill roles the maternal genes can’t, either by virtue of their cellular location or the timing of their expression (or simply because they were never intended to meet the later demands of development). These possibilities are supported by the finding that many zygotic genes show distinctive expression patterns at cycle 14, coinciding with the MZT. Since the expression levels for these genes had either stabilized or decreased during the MZT, the researchers concluded that maternal degradation and zygotic transcription occurred in tandem to influence their patterned expression.

The researchers go on to show that maternal transcripts and zygotic genes have distinct regulatory elements: one controls maternal transcript degradation, the other zygotic gene transcription. Activation of the zygotic genome—which accounts for roughly 20% of transcripts at cycle 14—begins when a protein deposited in the egg by the mother during oogenesis binds with the regulatory motif shared by the early zygotic genes.

This study raises a number of avenues of investigation, such as identifying the key regulatory genes responsible for calling the silent zygotic genome into action—a watershed moment for the developing embryo. Future studies can also explore whether the developmental patterns identified here hold true for other animals.

